# Evaluation of Quality of Care in Chagas Disease Cardiomyopathy

**DOI:** 10.5334/gh.1518

**Published:** 2026-02-03

**Authors:** Pablo Elías Gulayin, Maria-Jesus Pinazo, Rachel Marcus, Caryn Bern, Eva H. Clark, Maria Carmo Pereira Nunes, Bruno Ramos Nascimento, Shreya Shrikhande, Sean Taylor, Pablo Perel, Antonio Luiz Ribeiro

**Affiliations:** 1 Institute of Clinical Effectiveness and Health Policy (IECS), Argentina; 2 Drug for Neglected Diseases initiative, LATAM. Rio de Janeiro, Brazil; 3 Centro de Investigación Biomédica en Red de Enfermedades Infecciosas (CIBERINFEC), Instituto de Salud Carlos III, Madrid, Spain; 4 Latin American Society of Chagas, Washington DC, United States; 5 Department of Epidemiology and Biostatistics, University of California San Francisco, San Francisco CA, United States; 6 Departments of Medicine (Infectious Diseases) and Pediatrics (Tropical Medicine), Baylor College of Medicine, Houston, TX, United States; 7 Department of Internal Medicine, Faculdade de Medicina, and Telehealth Center and Cardiology Service, Hospital das Clínicas, Universidade Federal de Minas Gerais, Belo Horizonte, Brazil; 8 Serviço de Hemodinâmica, Hospital Madre Teresa, Belo Horizonte, MG, Brazil; 9 World Heart Federation, Geneva, Switzerland

**Keywords:** Chagas disease, quality of care, neglected disease, health system, Donabedian model

## Abstract

**Background:**

Chagas disease (ChD), a neglected tropical disease caused by *Trypanosoma cruzi*, affects around 7.5 to 10 million people globally, primarily in Latin America. Chronic Chagas cardiomyopathy (CCM) is the most severe clinical form, leading to substantial cardiovascular morbidity and mortality. Despite existing guidelines, fragmented health systems, low provider awareness, and limited access to care hinder effective disease management.

**Objectives:**

We aimed to define the key components of the CCM quality of care (structure, process, and outcomes) for main clinical activities at the three levels of care.

**Methods:**

We applied the Donabedian model to define essential components of ChD care at primary, secondary, and tertiary levels. Key recommendations from the World Heart Federation (WHF) roadmap and evidence-based guidelines were used to identify core services at each level. We also examined two case studies that demonstrate successful implementation of innovative screening and management models.

**Results:**

Essential components of ChD care were identified at all levels. Primary care plays a central role in early diagnosis and timely treatment. Secondary care addresses complications through imaging and targeted therapy, while tertiary care provides advanced interventions and rehabilitation. Although structural gaps persist, the implementation of systematic processes and clearly defined outcomes is key to strengthening the quality, continuity, and equity of care.

**Conclusions:**

A comprehensive, structured approach to ChD care is essential to improving outcomes. Successful models illustrate that scalable, resource-appropriate interventions can enhance diagnosis and treatment. Integration into routine health systems, supported by universal health coverage, improved data systems, and implementation research, is critical to closing the care gap and advancing equity in cardiovascular health.

## 1. Introduction

Chagas disease (ChD) remains a significant global health challenge, primarily affecting vulnerable populations in Latin America. Its burden is also increasing among immigrant communities in Europe and the United States (1,2). Despite substantial progress in controlling transmission through vector and transfusion-based mechanisms, millions of individuals continue to live with the disease. Chronic Chagas cardiomyopathy (CCM) is the most severe clinical form of ChD. It is defined by one or more typical electrocardiogram (ECG) abnormalities in seropositive individuals and is associated with severe complications (3,4). As a neglected tropical disease, ChD exemplifies persistent challenges in access to care in low-resource settings. Barriers to prevention, diagnosis, and treatment remain despite advances in public health interventions (5,6,7).

**Figure 1:**
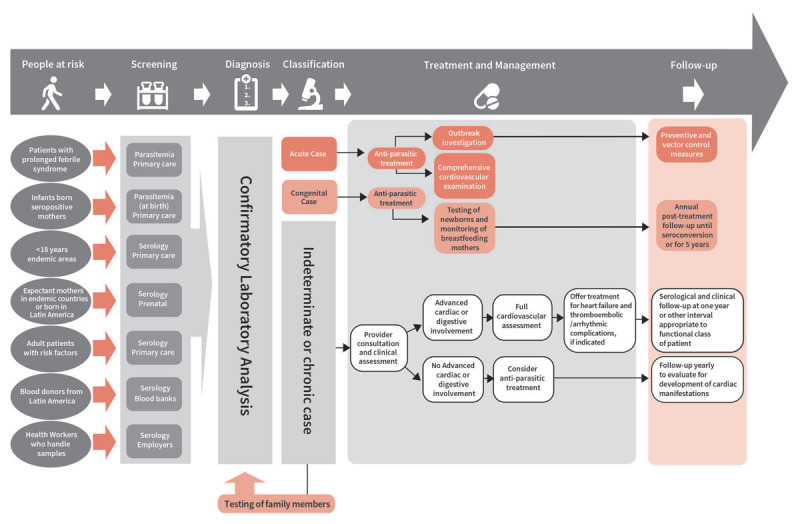
Ideal patient pathway for Chagas disease. Reproduced from the WHF IASC Roadmap on Chagas disease with permission (2).

In 2020, the World Heart Federation (WHF), along with the Inter-American Society of Cardiology (IASC), published a roadmap on ChD (2). The WHF-IASC Roadmap describes an ideal patient care pathway (Figure 1). It also proposes evidence-based strategies for healthcare professionals, health authorities, and governments to overcome barriers to comprehensive ChD care. Given the need to advance the implementation of these strategies, the WHF Neglected Cardiovascular Diseases Expert Group has prepared a document to help evaluate the quality of care for ChD patients, using the Donabedian framework (8). The Donabedian framework is a conceptual model used to evaluate healthcare quality. It also examines aspects of healthcare access across three dimensions: structure, process, and outcomes. The framework’s focus on system-level dimensions offers a structured approach to examining healthcare provision in both endemic and non-endemic settings, particularly for the management of chronic disease sequelae and cardiovascular care. In the present article, we aimed to define the key components of the CCM quality of care (structure, process, and outcomes) for ChD’s main clinical activities, considering primary, secondary, and tertiary healthcare levels. This analysis suggests actionable strategies to enhance healthcare access for populations affected by ChD globally. It highlights roadblocks within the patient care pathway and showcases successful experiences in overcoming these barriers.

## 2. Definitions and Methodological Considerations

In the 1960s, Avedis Donabedian introduced a framework for evaluating healthcare quality based on three key components: structure, process, and outcomes (9). “Structure” pertains to the environment in which care is provided, “process” focuses on the delivery of care, and “outcomes” reflect the impact on patient and population health. Since its inception, the Donabedian model has been widely adopted in healthcare quality assessments due to its adaptability to diverse clinical settings. It has been applied to enhance overall surgical quality and treatment of specific conditions, including lung and prostate cancer, congenital heart defects, and emergency general surgery (10).

Recent guidance documents (2,3,11) outline the core components of ChD management, beginning with the identification of patients with chronic infection, followed by their evaluation and treatment. Chronic ChD clinical manifestations range from asymptomatic patients with no or mild organic involvement, to severe heart or gastrointestinal disease. Cardiac manifestations are broad and include arrhythmias, dilated non-ischemic cardiomyopathy, and thromboembolic disease. Patients may need care at various levels of the health system depending on their disease stage. This article categorizes healthcare services into primary, secondary, and tertiary levels (12,13). Primary care is the first point of contact, addressing most medical needs, including treatment for acute and chronic conditions, preventive care, and health education. Care is typically managed by general practitioners, nurses, or physician assistants. Secondary care involves specialists in clinics or community hospitals for patients referred by primary care, such as specialized diagnostic modalities, treatment, or hospital-based care. Tertiary care encompasses highly specialized services provided by advanced hospitals, such as trauma care, organ transplants, or complex treatments for high-risk conditions. Strategies to strengthen the integration of primary, secondary, and tertiary care can help health systems respond to the increasing burden of chronic diseases and multimorbidity. Such integration may also reduce fragmentation of care delivered by multiple providers across different settings and levels (12,13).

This paper was developed through a consensus-driven approach coordinated by members of the WHF Neglected Cardiovascular Diseases Expert Group. The process involved collaboration with clinicians and researchers with expertise in ChD, cardiology, infectious diseases, and health systems. The identification of the core components of quality care for ChD at different levels of the health system was informed by a narrative review of existing guidance and evidence. This included the WHF/IASC Chagas Disease Roadmap, scientific statements from major cardiovascular societies, and WHO technical documents. We also reviewed peer-reviewed studies addressing ChD management, diagnostic tools, and health service delivery in both endemic and non-endemic settings. While the review was not systematic, it focused on high-quality and widely cited documents to capture current consensus and best practices.

The final adaptation of the Donabedian model for evaluating the quality of care in ChD cardiomyopathy was the result of multiple rounds of discussion among the co-authors and members of the WHF Neglected Cardiovascular Diseases Expert Group. Two case studies illustrating the successful implementation of screening and management models in low-resource settings were selected by consensus to highlight practical strategies for improving care quality and access. One example from an endemic country and one from a non-endemic country were intentionally included.

## 3. A Brief Summary of ChD

ChD is caused by the protozoan parasite *Trypanosoma cruzi* and transmitted through the infected feces of hematophagous triatomine vectors (3,11). Transmission occurs when infected vector feces enter through a break in the skin, such as the bite site, or intact conjunctiva or other mucosal surfaces. Vectorial transmission is often associated with rustic houses, especially those built of adobe or mud. Parasite transmission can also occur congenitally, through infected blood or organ donations, and via consumption of contaminated food or drink, notably sugarcane and açai derivatives.

Between 7.5 and 10 million people are currently living with *T. cruzi* infection, but most are unaware of their infection status (14,15). Many healthcare providers have low awareness of ChD and the need to screen those at highest risk, based on socioeconomic status, regional endemicity, and housing conditions (2,16). The earlier in the course of infection anti-trypanosomal treatment (i.e., benznidazole or nifurtimox) is accomplished, the more likely it is that end-organ damage will be prevented. Treatment of infected girls and women before they become pregnant is estimated to decrease the probability of congenital transmission by 95% (14). Active screening programs are crucial for detecting *T. cruzi* infection in individuals who can benefit from anti-trypanosomal treatment (2,11). This includes younger individuals, particularly those under 50 years of age, in the indeterminate phase or in the early stages of organ damage, for whom antiparasitic therapy may prevent morbidity or disease progression.

Acute infection tends to occur in childhood in endemic areas of Latin America. In these settings, the triatomine vector infests houses with adobe or mud walls and/or thatched roofs and proliferates in peridomestic environments such as poultry nests and animal corrals (11). The acute phase may be asymptomatic or cause mild, non-specific symptoms, such as fever, lymphadenopathy, and hepatosplenomegaly, but may include acute myocarditis, with a self-limited average duration of 4 to 8 weeks. Acute symptoms resolve without treatment in most *T. cruzi*-infected individuals; fewer than 1% are diagnosed during the acute phase, but without anti-trypanosomal treatment, they have high odds for developing lifelong chronic conditions. Antiparasitic therapy is highly effective during the acute phase, with parasitological cure rates of up to 80% (3); however its impact on chronic and advanced clinical forms remain controversial.

Most of those infected will remain asymptomatic for life—the so-called chronic indeterminate form of the disease—while approximately 30% progress to severe clinical chronic complications, with cardiomyopathy being the principal cause of incapacity and death (2,7,17). A smaller proportion develop gastrointestinal complications ChD or the mixed form, which combines both organ involvements. Patients with early clinical forms are good candidates for antiparasitic therapy (3). An updated meta-analysis suggested that antiparasitic treatment reduced ECG changes [risk ratio (RR): 0.48], disease progression (RR: 0.35), cardiovascular death (RR: 0.44), and overall mortality (RR: 0.54) compared to placebo or no intervention, although the risk of bias was moderate to high and a significant proportion of the studies included was observational (18).

CCM is pleomorphic. Clinically relevant manifestations include conduction system disturbances (typically right bundle branch or bifascicular block), sinus node dysfunction, atrioventricular blocks, and supraventricular and complex ventricular arrhythmias (3,11). Not uncommonly, patients may develop an aneurysm, most frequently in the apex of the left ventricle, a common source of thromboembolus. Dilated cardiomyopathy and congestive heart failure develop later in the course of the disease. This disease progression is associated with multiple pathways, mainly related to inflammation, fibrosis, autonomic impairment, and involvement of the conduction system. Patients with CCM have an increased risk of embolic strokes from thrombus in the aneurysm or dilated left ventricle. ChD is an independent predictor of this type of event among patients with stroke in endemic settings (19).

**Figure 2:**
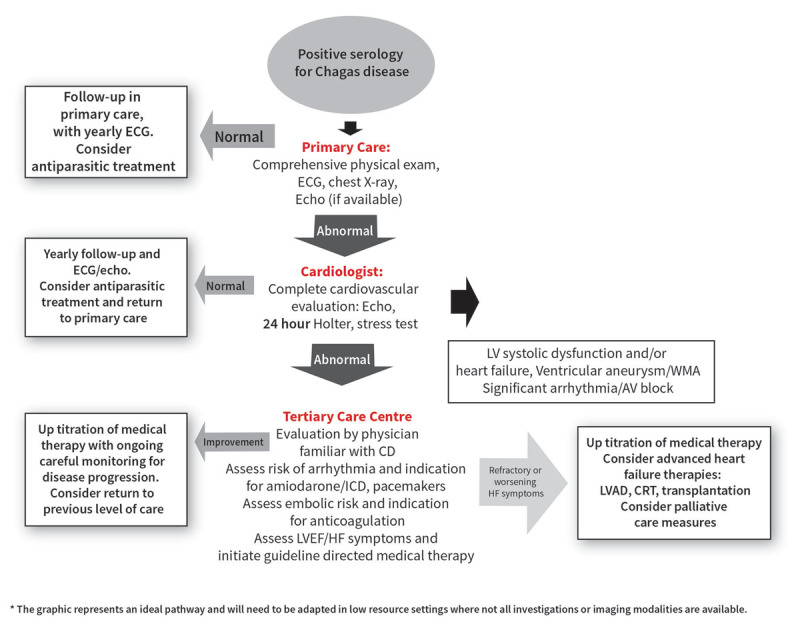
WHF roadmap recommendations for cardiovascular evaluation in patients with positive serology for ChD. Reproduced from the WHF IASC Roadmap on Chagas disease with permission (2).

Although anti-trypanosomal treatment failed to significantly decrease progression in a clinical trial of patients with established CCM, appropriate cardiac management prolongs survival and greatly improves the quality of life for patients with this clinical form (3,11,20). Treatment modalities include antiarrhythmics, pacemakers, intracardiac cardioverter-defibrillators (ICD), and medical management of congestive heart failure; however, a substantial proportion of existing recommendations extrapolate findings from studies involving other etiologies of heart disease, because CCM is critically underrepresented in clinical trials. Patients with end-stage CCM are candidates for transplantation and have survival figures equal to or better than those for patients transplanted for idiopathic dilated cardiomyopathy (21). Figure 2 summarizes the WHF roadmap recommendations for cardiovascular evaluation in patients with ChD.

## 4. The Donabedian Model of Healthcare Quality Applied to Healthcare in ChD

ChD requires comprehensive, lifelong care and management. This patient care approach ranges from primary to tertiary levels of treatment and intervention. However, given that ChD predominantly affects individuals in low-resource settings, an integrated strategy to optimize quality of care across different healthcare levels is essential for efficient management (2). It is important to highlight that patients with ChD often have comorbidities, which may further worsen their cardiac condition and place an additional burden on healthcare systems (11).

Timely diagnosis and treatment are critical for improving the quality of life and preventing disease progression. To ensure comprehensive management, all individuals affected by ChD should receive standardized services across the three previously described levels, guided by an evidence-based approach (2,11).

As CCM is the most common and severe complication, triage and early diagnosis at the primary healthcare level offer an opportunity for anti-trypanosomal treatment in the initial or early recognizable stages. This may prevent progression to severe structural involvement and advanced rhythm disturbances (2). Upon diagnosis, screening for clinical manifestations should include a detailed medical history and physical examination to identify signs and symptoms of chronic cardiac and gastrointestinal involvement (2,11). For all individuals with positive serology, an initial ECG should be performed to detect cardiac abnormalities and repeated regularly (e.g., annually or after the onset of new symptoms) to monitor disease progression. In individuals with a normal ECG, new changes indicate progression to the cardiac form of the disease, warranting further diagnostic testing and referral to a cardiologist at a specialized care center. After specialist evaluation, patients with mild abnormalities may be eligible for regular follow-up at the primary care level. Reassessment at a higher level of care should be considered if major abnormalities or new symptoms emerge.

In secondary care settings, an initial echocardiogram is recommended for individuals with an abnormal ECG to assess the severity of cardiac disease and guide patient management. According to international guidelines and recommendations, the echocardiogram should be regularly repeated, preferably every 1–2 years (3), if ventricular dysfunction is present or if new cardiovascular symptoms develop (22). Although most clinical evidence derives from trials involving other etiologies of heart failure, guideline-directed medical therapy should be initiated as appropriate in patients with left ventricular systolic dysfunction. This typically includes beta-blockers, angiotensin-converting enzyme inhibitors or angiotensin receptor blockers, angiotensin receptor–neprilysin inhibitors, mineralocorticoid receptor antagonists, and sodium-glucose cotransporter-2 inhibitors (SGLT2i) (2,23). In addition, arrhythmia risk should be evaluated with a 24-hour Holter monitor or an exercise test to detect exercise-induced ventricular arrhythmias and chronotropic incompetence (2,3,11). Risk stratification for embolic events is also crucial, as stroke is a common clinical manifestation, irrespective of the severity of CCM. Although challenging, this risk evaluation may be conducted through routine reassessment of rhythm disturbances and echocardiographic monitoring for new onset of structural abnormalities, notably apical aneurysms and ventricular thrombi.

Finally, in cases of clinical deterioration, patients should be referred to a tertiary care center with advanced heart failure treatment options, including device therapies, catheter ablation of arrhythmias, and heart transplantation. Referral to the third level is also necessary if electrophysiological treatment of complex arrhythmias is required.

Comprehensive access to quality of care is an important challenge for people at risk of *T. cruzi* infection in both endemic and non-endemic settings. Individuals with ChD are generally impoverished, uninsured, and are not able to manage their chronic condition. To receive appropriate care, individuals must have access to initial evaluations and treatments at no or low cost. This assumption remains true in non-endemic countries, where advanced medical care is more widely available but is often inaccessible to the typical patient afflicted with ChD.

Additionally, individuals at risk of *T. cruzi* infection in non-endemic countries, where ChD prevalence has increased, may face multiple barriers. These include vulnerable social conditions (e.g., migratory status, lack of health insurance, insufficient income) and low clinician awareness of ChD (14,24,25). In some endemic countries, patients with known ChD may have better access to initial clinical evaluation but face limited access to more complex studies, such as echocardiograms and other tests needed to evaluate the clinical progression of *T. cruzi* infection. These limitations in quality of care are not merely a matter of prioritization; overall knowledge and education about ChD, in all its complexity, remain limited, particularly among healthcare providers and policy makers.

Table 1 presents the minimum structure, the processes to be performed, and the outcomes to be assessed according to the different stages of the disease and the level of care, using the previously described Donabedian framework. This integrated approach is especially important in non-endemic countries, where the disease is less recognized and poorly understood.

**Table 1:** Description of types of care typically provided at primary, secondary, and tertiary facilities.


LEVEL OF CARE	STRUCTURE	PROCESS	OUTCOMES

All levels	Training materials for ChD management.	Regular training of healthcare teams on ChD detection, evaluation, and management.	Proportion of trained healthcare professionals.

All levels	Electronic medical records.	Systematic documentation of ChD cases in EMRs.	Accuracy of EMRs identification of ChD cases.

Primary	Serological tests for ChD detection.	Guideline-based screening for ChD in endemic and non-endemic settings.	Rate of confirmed diagnoses with two positive serological tests.

Primary Secondary	ECG and chest X-ray.	Annual assessment for early detection and risk stratification of cardiac complications.	Incidence of new cardiac abnormalities.

Secondary	Echocardiography.	Guideline-based echocardiographic follow-up.	Incidence of new echocardiographic abnormalities

Primary Secondary	Guideline-directed medical therapy for specific organ complications.	Assessment of organ involvement to guide initiation of GDMT.	Proportion receiving GDMT when indicated.

Primary Secondary	Antiparasitic treatment.	Guideline-based prescription and monitoring of antiparasitic therapy.	Antiparasitic treatment completion rate.

Secondary	Specialists for antiparasitic treatment follow-up.	Follow-up of adverse drug reactions requiring specialist care.	Rate of adverse reactions to antiparasitic treatment requiring specialist care.

Secondary Tertiary	Specialized cardiac studies (24-hour Holter monitoring, stress test, advanced cardiovascular imaging).	Specialist cardiac evaluation and follow-up in patients with moderate-to-severe CCM.	Completion rate of indicated advanced cardiac studies.

Secondary Tertiary	Specialized gastroenterological studies (barium enema and barium swallow).	Specialist evaluation and follow-up of gastrointestinal involvement.	Proportion undergoing specialized GI evaluation when indicated.

Secondary Tertiary	Advanced cardiac therapies/interventions (pacemaker, ICD, ventricular tachycardia ablation, LVAD, heart transplantation).	Delivery of advanced cardiac therapies/interventions for moderate-to-severe CCM.	Utilization rate of advanced cardiac therapies/interventions when indicated.

Secondary Tertiary	Resources and trained personnel for cardiac rehabilitation.	Guideline-based referral to cardiac rehabilitation programs.	Proportion of patients completing indicated cardiac rehabilitation.

Secondary Tertiary	Quantitative PCR devices and trained personnel knowledgeable in ChD treatment and severity criteria.	Early detection and management of ChD reactivation in immunosuppressed patients.	Treatment rate for confirmed disease reactivation.


ChD: Chagas disease; EMR: Electronic medical record; ECG: electrocardiogram; GDMT: Guideline-directed medical therapy; CCM: Chronic Chagas cardiomyopathy; ICD: implantable cardioverter-defibrillator; LVAD: left ventricular assist device.

## 5. Examples of Successful Experiences

### Example #1: Improving access to screening for ChD in Harris Health, Houston, Texas, United States

Goal: Detect and treat chronic ChD before the development of cardiac sequelae.

One of the most effective strategies to prevent CCM is through early intervention. Early detection of ChD and timely treatment of eligible patients with anti-trypanosomal therapy, such as benznidazole or nifurtimox, are critical. The earlier treatment is administered following *T. cruzi* infection—particularly during childhood or soon after exposure—the lower the individual’s risk of developing CCM. Even when patients are not eligible for anti-trypanosomal treatment due to age or established structural heart disease, early detection of CCM enables timely referral for cardiology management. To improve early detection of ChD and the diagnosis of its complications, many institutions in the Americas have implemented systematic screening initiatives. These programs aim to be easily accessible and low-cost. The program developed within Harris Health (HH) in Houston, Texas, exemplifies a successful ChD screening initiative in a low-resource healthcare system serving uninsured and underinsured residents of Harris County, Texas.

The HH screening program began to be developed in 2021 as part of a research program to identify the best screening approach for people living with HIV with risk factors for ChD (26). It continues to evolve as part of a quality improvement project encompassing five HH primary care clinics: Internal Medicine, Internal Medicine/Pediatrics, Family Medicine, HIV Obstetric, and HIV Primary Care.

An initial challenge was the limited knowledge of ChD among frontline clinicians, as it is not routinely covered in health professional training in the United States. We documented a significant improvement in clinicians’ knowledge of the ChD after holding hour-long training sessions tailored to the needs of the clinicians at each clinic.

A second challenge was coordinating a ChD screening and confirmatory testing algorithm with the HH clinical laboratory. The final testing protocol follows the US Centers for Disease Control and Prevention (CDC) guidelines and is as follows: the clinician places an order for the Hemagen IgG ELISA (LabCorp/ARUP), which serves as the initial screening test. If the result is positive, saved serum is sent to the Texas Department of State Health Services (TX DSHS) for a repeat of the Hemagen assay and confirmatory testing via the Wiener IgG ELISA. If the results are discordant, a third serological test should be obtained. A patient is considered to have confirmed ChD if any two distinct tests are positive.

A third challenge was the limited awareness among busy clinicians regarding the need to screen their patients for ChD. To address this barrier, we implemented several strategies, including:

- Training nursing staff in charge of HIV “entry-to-care” visits to include a ChD screening test as part of their usual lab panel for patients who reported residence for at least 6 months in continental Latin America;
- Developing an algorithm for prenatal screening of pregnant people living with HIV that includes ChD screening for those at risk—notably immigrants from Latin American countries – and, if confirmatory testing is positive, a plan to notify neonatology so that the infant is appropriately tested for congenital ChD at delivery and followed up with Pediatric Tropical Medicine;
- Training the triage nursing staff at the Internal Medicine/Pediatrics clinic to use an electronic health record (EHR; in this case, Epic) “smart phrase” to document whether the patient had resided for at least 6 months in continental Latin America; viewing the nursing note then triggers the clinician to order the screening test for patients who answered positively;
- Training clinicians to include a reminder in their Hnote templates—standardized layouts used to guide complete patient-intake documentation—to ensure screening of at-risk patients;
- Assigning one Infectious Diseases/Tropical Medicine clinician to review the results of all the ChD screening tests sent within HH monthly to ensure that patients who screen positive are referred to Infectious Diseases/Tropical Medicine; and
- Creating an “Immigrant Health Screening” EHR order panel, accessible to all outpatient HH clinicians, that includes orders for ChD serology for patients who have resided in continental Latin America for more than 6 months.

Once a patient is confirmed to have ChD by having two positive tests of distinct types per Pan American Health Organization (PAHO) guidelines, either the frontline clinician or the Infectious Diseases/Tropical Medicine physician is responsible for screening the patient for cardiac, gastrointestinal, and neurological symptoms, as well as sending baseline cardiac tests (ECG and transthoracic echocardiogram), and referring to additional HH subspecialists, such as cardiology or gastroenterology, if warranted. The Infectious Diseases/Tropical Medicine physician is responsible for counselling the patient on ChD, evaluating whether the patient is eligible for anti-trypanosomal treatment, prescribing anti-trypanosomal treatment if the patient is eligible and monitoring the patient during treatment, and offering screening to the patient’s at-risk family members.

This work has been successful, as demonstrated by a significant rise in ChD monthly screening rates, increasing from 13 *T. cruzi* IgG screening tests sent in July 2023 to 109 sent in March 2025. The HH ChD screening program is an example of how screening can be implemented successfully in a large, low-resource, sliding-scale US health system via a quality improvement project, without external funding or significantly increased costs on the part of the patient. The HH has a number of advantages over other healthcare systems serving similar populations: it is relatively well supported by the local Harris County government; is well-established and trusted by the local population including both documented and undocumented US residents; has extensive infrastructure including two large tertiary care hospitals and many outpatient subspecialty clinics stretching throughout Harris County; boasts an easy-to-use EHR; and is led by a team open to new ideas and screening/prevention programs that will benefit its patients.

### Example #2: Development of an artificial intelligence (AI)-based model for detecting ChD in cardiovascular screening programs in Brazilian primary care

Goal: To build a model for implementing AI-based diagnosis of ChD, based on tele-ECG and simplified clinical questionnaires, and to implement echocardiographic screening in primary care through task-shifting and telemedicine support.

In Brazil, there is limited access to specialized care, including cardiology referrals and specific tests, such as the standard echocardiogram. This leads to long waiting queues, often without prioritization strategies to identify severe cases at the highest risk. Although ultrasound imaging in Brazil can only be performed by certified physicians, a task-shifting strategy using handheld echocardiography was first tested in 2014 within research protocols. This approach was supported by remote expert interpretation through telemedicine platforms and implemented as part of a rheumatic heart disease screening program (PROVAR+) (27). The initial approach, based on screening of school children and teenagers, was successful and was expanded to primary care in 2017, with the aim of diagnosing structural heart disease early and prioritizing referrals. Non-physicians from different backgrounds underwent supervised training using simplified imaging protocols. They were able to identify ventricular involvement, valvular disease, and other forms of structural abnormalities for triage purposes (28). This strategy was well-received during its implementation phase and resulted in the detection of a large burden of hidden cardiac disease, with high sensitivity (but suboptimal specificity) for identifying and prioritizing the most severe cases (29,30). The program, initially focused on some areas of the state of Minas Gerais, has since been expanded to other regions, including the Northeast. More recently, tele-ECG has been incorporated as part of the triage process, with automated flagging of individuals exhibiting major abnormalities for echocardiographic screening. This approach has also proven feasible, with major ECG abnormalities detected in screening echocardiograms being associated with an over 2-fold increase in the risk of having major heart disease (31). Thus, the successful implementation of such strategies promises to improve the diagnosis of CCM and the prioritization of care for those with advanced cardiac sequelae.

Furthermore, the coverage of the Telehealth Network of Minas Gerais—a network of public institutions that provides telemedicine resources, including the tele-ECG, to over 1,500 cities in Brazil and South America—could be leveraged by including AI-powered ECG (AI-ECG) triage solutions that have been developed for the diagnosis of ChD. An algorithm that utilizes the tele-AI-ECG and simple clinical data (housing conditions, family history of ChD, and contact with the triatomine bug) to predict the disease has shown promising results, with an area under the ROC (Receiver Operating Characteristic) approaching 0.70 in validation datasets from endemic settings, and reaching 0.77 and 0.80 for patients with a diagnosis of CCM in outpatient cohorts (32). More recently, this AI-ECG triage was implemented in a joint echocardiogram screening effort in a low-resourced municipality of Bahia, Northeast Brazil, with the trigger system at the point of care. A 100% sensitivity was observed at the cost of 40% specificity, resulting in a diagnostic odds ratio of 6.6 (33). The tool is now being widely tested in the nationwide tele-ECG system and is a promising tool for early diagnosis, especially where there is a lack of experts. Furthermore, the potential of predicting ventricular dysfunction in patients with a diagnosis of ChD from AI-interpreted ECG alone is under investigation. Currently available data show an accuracy of over 80% in high-prevalence samples (34).

## 6. Challenges and Perspectives

Improving quality care for ChD remains a major global health challenge. Despite progress in vector control and increased awareness, the disease continues to disproportionately affect low-income, marginalized populations, both in endemic and non-endemic regions (1). Additionally, although infection rates have decreased in the past decades, advanced sequelae have shifted to older ages, posing additional challenges and costs to health systems (14). Structural barriers, limited diagnostic and therapeutic coverage, low awareness of the disease among healthcare providers and the general population, and insufficient coordination within the health system compromise care delivery throughout the patient journey—from screening and diagnosis to specialized cardiac interventions (2).

### Fragmented systems and the imperative of universal health coverage

Universal health coverage (UHC) is a necessary condition for delivering adequate care for ChD (2). Without guaranteed access through publicly funded systems, patients are unlikely to receive the timely diagnostic and therapeutic services they require. ChD affects historically excluded populations—low-income individuals in rural endemic areas, migrants in non-endemic countries, and people with limited or no insurance—who depend on public systems for their healthcare. When these systems are fragmented, underfunded, or exclude critical services from their benefit packages, comprehensive care becomes inaccessible.

The care pathway for ChD requires more than a one-time intervention; ensuring continuity along the continuum of care—from screening and diagnosis to long-term management and advanced interventions—is essential to address the progressive nature of ChD and to avoid fragmentation and loss to follow-up. This depends on access to triage and risk stratification routines, serological diagnosis, antiparasitic treatment, and serial ECGs and echocardiograms. It also requires arrhythmia risk stratification, cardiac rehabilitation, and, when indicated, access to invasive diagnostic and therapeutic modalities, including implantable devices or heart transplantation. Making these services available at the appropriate level of care—primary, secondary, and tertiary—is essential. As detailed in Table 1, this requires investments in infrastructure, equipment, and trained personnel appropriate to each level. However, availability alone is insufficient: integration across levels is what ensures continuity, prevents duplication, and enables timely referral and management (12).

Strengthening UHC for ChD involves ensuring the public provision of, or reimbursement for, essential services at every stage of the disease. It also requires expanding coverage to triage and diagnostic tools (e.g., ECG, imaging), guideline-driven medical therapy, and access to specialist evaluation. Without UHC, isolated programs, no matter how well designed, will have limited reach and sustainability.

### Implementation, scaling-up, and sustainability

Despite well-documented strategies for the prevention, diagnosis, and management of ChD, a substantial gap remains between evidence and practice. Effective local initiatives – such as provider-based screening integrated into EHRs in the United States or AI-ECG opportunistic screening in Brazil—are rarely scaled beyond pilot phases. This reflects limited political commitment, scarce funding, and weak institutional mechanisms for translating innovation into policy.

Sustainable implementation requires embedding ChD care into routine health services, supported by investments in human resources, supply chains, and referral systems. Task-shifting strategies (35), such as implementing echocardiogram imaging by trained technicians or empowering primary care teams to manage the early stages of the disease, have proven effective in other neglected conditions (36) and are applicable here. Digital health tools, such as teleconsultation, remote ECG and echocardiogram interpretation, and mobile health platforms (29,37,38), can also extend service reach, but must be adapted to low-connectivity areas and populations with limited digital literacy.

To support scale-up, interventions must be monitored for quality, cost-effectiveness, and equity. Strengthening country-level governance and planning mechanisms is essential for aligning local delivery with national strategies and international targets.

### Data systems and performance monitoring

The absence of robust monitoring systems limits the ability to track patient outcomes and evaluate program effectiveness. ChD is often poorly integrated into national health information systems, and key indicators—such as treatment completion, disease progression, or complications—are rarely recorded in a standardized manner. Strengthening electronic medical records systems is therefore highly relevant, as it enables the systematic identification of ChD patients and the implementation of automated alerts for pending evaluations, which can substantially improve quality-of-care monitoring, reduce loss to follow-up, and support timely clinical decision-making.

Surveillance systems are also fragmented, and essential statistics often underreport ChD-related mortality. Compulsory notification of at least acute cases to national surveillance systems, together with the establishment of a national registry of ChD patients, could play a fundamental role in highlighting the true burden of the disease (39,40). To improve the quality of care and guide policy, countries need interoperable digital platforms capable of integrating data from primary care, hospitals, and laboratories. The Donabedian framework, used in this article, offers a roadmap for defining and tracking structure, process, and outcome indicators across care levels.

### Local research

Despite its high burden of disease, ChD is one of the most neglected tropical diseases, and research funding remains limited (41). Most therapeutic recommendations are based on extrapolation from other conditions or are derived from observational studies. There is an urgent need for pragmatic trials, implementation research, and operational studies tailored to different health system contexts and integrated with existing healthcare strategies.

Local research capacity should be strengthened to allow the evaluation of simplified care models and the optimization of resource use, with findings that can subsequently inform national strategies. Community-based studies are particularly important for assessing barriers to care, treatment adherence, and social determinants of health. Moreover, affected countries should be active participants—not merely recipients—in international research collaborations and trials involving novel antiparasitic drugs, cardiac interventions, and digital health solutions (42).

### A path forward

ChD illustrates how health inequities translate into preventable suffering. Addressing this issue requires more than clinical guidelines; it demands structural reform, integration into UHC frameworks, investment in frontline care, and the generation of locally generated knowledge. Strengthening data systems, institutionalizing monitoring, and scaling up proven strategies are key to ensuring that high-quality care reaches those most in need. Ultimately, ChD care must become a routine part of health systems, rather than an exception, if we are to achieve the vision of cardiovascular health for all.
